# 119 Exercise in the ICU and its Effects on Physical and Mental Health in Pediatric Burn

**DOI:** 10.1093/jbcr/irad045.092

**Published:** 2023-05-15

**Authors:** Andrea Rego, Ingrid Parry, Soman Sen, Heidi Spratt, Jong Lee, David Herndon, Steven Wolf, Ludwik Branski, Oscar Suman-Vejas

**Affiliations:** The University of Texas Medical Branch, Galveston, Texas; Shriners Hospital for Children, Northern California, Carmichael, California; Shriners Hospital for Children Northern California, Sacramento, California; University of Texas Medical Branch, Galveston, Texas; The University of Texas Medical Branch, Galveston, Texas; CEO, Joseph Still Burn Research Foundation, Senior Editor Journal of Burn Care and Research, Augusta, Georgia; The University of Texas Medical Branch, Galveston, Texas; Shriners Children's- Texas, University of Texas Medical Branch, Galveston, Texas; The University of Texas Medical Branch, Galveston, Texas; The University of Texas Medical Branch, Galveston, Texas; Shriners Hospital for Children, Northern California, Carmichael, California; Shriners Hospital for Children Northern California, Sacramento, California; University of Texas Medical Branch, Galveston, Texas; The University of Texas Medical Branch, Galveston, Texas; CEO, Joseph Still Burn Research Foundation, Senior Editor Journal of Burn Care and Research, Augusta, Georgia; The University of Texas Medical Branch, Galveston, Texas; Shriners Children's- Texas, University of Texas Medical Branch, Galveston, Texas; The University of Texas Medical Branch, Galveston, Texas; The University of Texas Medical Branch, Galveston, Texas; Shriners Hospital for Children, Northern California, Carmichael, California; Shriners Hospital for Children Northern California, Sacramento, California; University of Texas Medical Branch, Galveston, Texas; The University of Texas Medical Branch, Galveston, Texas; CEO, Joseph Still Burn Research Foundation, Senior Editor Journal of Burn Care and Research, Augusta, Georgia; The University of Texas Medical Branch, Galveston, Texas; Shriners Children's- Texas, University of Texas Medical Branch, Galveston, Texas; The University of Texas Medical Branch, Galveston, Texas; The University of Texas Medical Branch, Galveston, Texas; Shriners Hospital for Children, Northern California, Carmichael, California; Shriners Hospital for Children Northern California, Sacramento, California; University of Texas Medical Branch, Galveston, Texas; The University of Texas Medical Branch, Galveston, Texas; CEO, Joseph Still Burn Research Foundation, Senior Editor Journal of Burn Care and Research, Augusta, Georgia; The University of Texas Medical Branch, Galveston, Texas; Shriners Children's- Texas, University of Texas Medical Branch, Galveston, Texas; The University of Texas Medical Branch, Galveston, Texas; The University of Texas Medical Branch, Galveston, Texas; Shriners Hospital for Children, Northern California, Carmichael, California; Shriners Hospital for Children Northern California, Sacramento, California; University of Texas Medical Branch, Galveston, Texas; The University of Texas Medical Branch, Galveston, Texas; CEO, Joseph Still Burn Research Foundation, Senior Editor Journal of Burn Care and Research, Augusta, Georgia; The University of Texas Medical Branch, Galveston, Texas; Shriners Children's- Texas, University of Texas Medical Branch, Galveston, Texas; The University of Texas Medical Branch, Galveston, Texas; The University of Texas Medical Branch, Galveston, Texas; Shriners Hospital for Children, Northern California, Carmichael, California; Shriners Hospital for Children Northern California, Sacramento, California; University of Texas Medical Branch, Galveston, Texas; The University of Texas Medical Branch, Galveston, Texas; CEO, Joseph Still Burn Research Foundation, Senior Editor Journal of Burn Care and Research, Augusta, Georgia; The University of Texas Medical Branch, Galveston, Texas; Shriners Children's- Texas, University of Texas Medical Branch, Galveston, Texas; The University of Texas Medical Branch, Galveston, Texas; The University of Texas Medical Branch, Galveston, Texas; Shriners Hospital for Children, Northern California, Carmichael, California; Shriners Hospital for Children Northern California, Sacramento, California; University of Texas Medical Branch, Galveston, Texas; The University of Texas Medical Branch, Galveston, Texas; CEO, Joseph Still Burn Research Foundation, Senior Editor Journal of Burn Care and Research, Augusta, Georgia; The University of Texas Medical Branch, Galveston, Texas; Shriners Children's- Texas, University of Texas Medical Branch, Galveston, Texas; The University of Texas Medical Branch, Galveston, Texas; The University of Texas Medical Branch, Galveston, Texas; Shriners Hospital for Children, Northern California, Carmichael, California; Shriners Hospital for Children Northern California, Sacramento, California; University of Texas Medical Branch, Galveston, Texas; The University of Texas Medical Branch, Galveston, Texas; CEO, Joseph Still Burn Research Foundation, Senior Editor Journal of Burn Care and Research, Augusta, Georgia; The University of Texas Medical Branch, Galveston, Texas; Shriners Children's- Texas, University of Texas Medical Branch, Galveston, Texas; The University of Texas Medical Branch, Galveston, Texas; The University of Texas Medical Branch, Galveston, Texas; Shriners Hospital for Children, Northern California, Carmichael, California; Shriners Hospital for Children Northern California, Sacramento, California; University of Texas Medical Branch, Galveston, Texas; The University of Texas Medical Branch, Galveston, Texas; CEO, Joseph Still Burn Research Foundation, Senior Editor Journal of Burn Care and Research, Augusta, Georgia; The University of Texas Medical Branch, Galveston, Texas; Shriners Children's- Texas, University of Texas Medical Branch, Galveston, Texas; The University of Texas Medical Branch, Galveston, Texas

## Abstract

**Introduction:**

Suffering a severe burn is an event that can have lifelong physical and mental ramifications. Exercise after discharge from the hospital, has been shown to improve quality of life with better physical and mental status in children with severe burns. However, the effect of exercise, implemented in the acute care setting, on quality of life in the specific population of adolescent patients with burns needs more research. The purpose of this study was to determine the effects of aerobic exercising in the burn intensive care unit (BICU) on patient self-reported physical and mental health outcomes in severely burned children. We hypothesized that aerobic exercise would improve self-reported physical and mental outcomes.

**Methods:**

This was a multi-center, prospective, randomized controlled trial. 48 children between the ages of seven and seventeen with ≥ 30% total body surface area (TBSA) burn were randomized in a 1:2 study design to standard of care (SOC) or standard of care plus receiving additional exercise with a cycle ergometer (SOC+Ex). Self-reported physical and mental health outcomes were obtained via surveys at admission and at discharge from the BICU.

**Results:**

Pain, weakness, and tiredness scores were significantly decreased from admission to the discharge time point in both groups (p< 0.05). Linear regression modeling revealed that there were no differences in pain, weakness, and tiredness levels due to treatment at any timepoint between groups. When controlling for age, gender, length of stay, number of exercise sessions, and %TBSA, time point makes a difference in pain, weakness, and tiredness levels (p < 0.0001). Sleep, sadness and misery scores were significantly decreased from admission to the discharge time point in both groups (p< 0.05). When controlling for age, gender, length of stay, number of exercise sessions, and %TBSA, time made a difference in sleep, sadness and miserable levels (p < 0.0001). There was a significant interaction (p = 0.0285) between time and treatment for the misery score.

**Conclusions:**

The findings of this study suggest that aerobic exercise in the BICU does not reduce self-reported physical and mental outcomes for severely burned children. Our recommendation is for all pediatric patients in the BICU to continue with the SOC and consult with their physician over the benefits of additional aerobic exercise. This study suggests that perhaps there is potential for increasing the amount of exercise that can be administered to pediatric burn survivors beyond SOC as we did not find aerobic exercise to result in any adverse events or to be of any harm to the patients if it is performed properly and under supervision.

**Applicability of Research to Practice:**

Additional aerobic exercise in the acute care setting is beneficial for severely burned children in terms of self-reported physical and mental health outcomes.

